# Clinical and hematological effects of hydroxyurea therapy in sickle cell patients: a single-center experience in Brazil

**DOI:** 10.1590/1516-3180.2013.1314467

**Published:** 2013-08-01

**Authors:** Ana Cristina Silva-Pinto, Ivan Lucena Angulo, Denise Menezes Brunetta, Fabia Idalina Rodrigues Neves, Sarah Cristina Bassi, Gil Cunha De Santis, Dimas Tadeu Covas

**Affiliations:** I MD, PhD. Associate Hematologist and Coordinator of the Sickle Cell Program, Centro Regional de Hemoterapia de Ribeirão Preto, Department of Internal Medicine, Faculdade de Medicina de Ribeirão Preto (FMRP), Universidade de São Paulo (USP), São Paulo, Brazil.; II MD, PhD. Associate Hematologist, Centro Regional de Hemoterapia de Ribeirão Preto, Department of Internal Medicine, Faculdade de Medicina de Ribeirão Preto (FMRP), Universidade de São Paulo (USP), São Paulo, Brazil.; III MD. Associate Hematologist, Centro Regional de Hemoterapia de Ribeirão Preto, Department of Internal Medicine, Faculdade de Medicina de Ribeirão Preto (FMRP), Universidade de São Paulo (USP), São Paulo, Brazil.; IV MD. Associate Hematologist, Department of Internal Medicine, Faculdade de Medicina de Ribeirão Preto (FMRP), Universidade de São Paulo (USP) São Paulo, Brazil.; V MD, PhD. Professor, Department of Internal Medicine, Faculdade de Medicina de Ribeirão Preto (FMRP), Universidade de São Paulo (USP) São Paulo, Brazil.

**Keywords:** Anemia, sickle cell. Hydroxyurea. Fetal hemoglobin. Acute chest syndrome. Erythrocyte indices, Anemia falciforme, Hidroxiuréia, Hemoglobina fetal, Síndrome torácica aguda, Índices de eritrócitos

## Abstract

**CONTEXT AND OBJECTIVES:**

Sickle cell disease (SCD) is the most common genetic disorder among people of African descent, affecting approximately 3,500 newborns each year in Brazil. Hydroxyurea (HU) is the only effective drug to treating patients with SCD, thereby reducing morbidity and mortality. The objective was to analyze the effects of HU on SCD patients at our institution.

**DESIGN AND SETTING:**

Retrospective study conducted at a sickle cell centre in Ribeirão Preto, São Paulo, Brazil.

**METHODS:**

We analyzed clinical and laboratory data on 37 patients. The hematological parameters and clinical events that occurred during the year before and the first year of treatment with HU were analyzed. The mean dose of HU was 24.5 ± 5.5 mg/kg/day.

**RESULTS:**

There were rises in three parameters: hemoglobin (8.3 g/dl to 9.0 g/dl, P *=* 0.0003), fetal hemoglobin (HbF) (2.6% to 19.8%, P *<* 0.0001) and mean cell volume MCV (89 to 105 fl, P *=* 0.001); and reductions in the numbers of leukocytes (10,050/µl to 5,700/µl, P *<* 0.0001), neutrophils (6,200/µl to 3,400/µl, P *=* 0.001), platelets (459,000/µl to 373,000/µl, P *=* 0.0002), painful crises (1.86 to 0.81, P = 0.0014), acute chest syndromes (0.35 to 0.08, P = 0.0045), infections (1.03 to 0.5, P = 0.047), hospitalizations (1.63 to 0.53, P = 0.0013) and transfusions (1.23 to 0.1, P = 0.0051).

**CONCLUSION:**

The patients presented clinical and hematological improvements, with an increase in HbF and a reduction in the infection rate, which had not been addressed in most previous studies.

## RESUMO

**CONTEXTO E OBJETIVO:**

A doença falciforme (SCD) é o distúrbio genético mais comum entre afrodescendentes, afetando aproximadamente 3.500 recém-nascidos a cada ano no Brasil. A hidroxiureia (HU) é a única droga efetiva para o tratamento dos pacientes com SCD, reduzindo a morbidade e a mortalidade da doença. O objetivo do estudo foi analisar os efeitos da HU em pacientes com SCD em nossa instituição.

**TIPO DE ESTUDO E LOCAL:**

Estudo retrospectivo realizado em um centro de anemia falciforme em Ribeirão Preto, São Paulo, Brasil.

**MÉTODOS:**

Nós analisamos os dados clínicos e laboratoriais de 37 pacientes. Os parâmetros hematológicos e eventos clínicos que ocorreram no ano anterior e durante o primeiro ano de tratamento com HU foram analisados. A dose média de HU foi 24.5 ± 5.5 mg/kg/dia.

**RESULTADOS:**

Houve aumento em três parâmetros estudados: hemoglobina (8,3 g/dl para 9,0 g/dl, P *=* 0,0003), hemoglobina fetal (HbF) (2,6% para 19,8%, P *<* 0,0001) e volume corpuscular médio (VCM) (89 para 105 fl, P *=* 0,001); e redução do número de leucócitos (10.050/µl para 5.700/µl, P *<* 0,0001), neutrófilos (6.200/µl para 3.400/µl, P *=* 0,001), plaquetas (459.000/µl para 373.000/µl, P *=* 0,0002), crises dolorosas (1,86 para 0,81, P = 0,0014), síndrome torácica aguda (0,35 para 0,08, P = 0,0045), infecções (1,03 para 0,5, P = 0,047), hospitalizações (1,63 para 0,53, P = 0,0013) e número de transfusões (1,23 para 0,1, P = 0,0051).

**CONCLUSÃO:**

Os pacientes apresentaram melhora clínica e hematológica, com aumento da HbF e redução da taxa de infecção, dado este não explorado na maioria dos estudos clínicos já publicados.

## INTRODUCTION

Sickle cell disease (SCD) is the most common genetic disorder among people of African descent, affecting approximately 3,500 newborns each year in Brazil. The underlying abnormality is a single nucleotide substitution (GTG for GAG) in the gene that encodes the b-globin chain located on chromosome 11. The mutated globin chain will form the abnormal hemoglobin S (HbS) seen in sickle cell patients. Upon deoxygenation, HbS molecules polymerize and change the red cell conformation into sickle-shaped cells.^1^ These cells are more adherent to the endothelium and to other cells, thus leading to hemolysis and vaso-occlusion, which are known to be painful episodes.^2^

Hydroxyurea (HU) is currently the only effective drug for treating patients with SCD, thereby reducing morbidity and mortality.^3^^,^^4^^,^^5^ The first randomized multicenter study that proved the efficacy of HU therapy among sickle cell patients (MSH), which was conducted in the 1990s, had a major impact on the management of sickle cell disease. It showed that HU can reduce painful episodes, length of hospital stay and number of red blood cell (RBC) transfusions, and can provide a 50% reduction in the occurrence of new episodes of acute chest syndrome (ACS).^6^

Nevertheless, the mechanisms through which HU exerts its clinical benefits in SCD cases are only partially known. The drug enhances the production of fetal hemoglobin (HbF), which decreases the polymerization of hemoglobin S (HbS),^7^^,^^8^ blocks the formation of sickle erythrocytes and prevents vaso-occlusive crises (VOC).^6^^,^^9^^,^^10^ However, clinical improvement occurs before the increment in HbF, thus suggesting that concurrent mechanisms may exist.^10^^,^^11^^,^^12^ These include (i) reduction of the number of white cells, platelets and reticulocyte counts; (ii) reduction of sickle cell adhesiveness mediated by a lower expression of surface adhesion molecules; (iii) induction of nitric oxide production (NO); and (iv) increase in the cell volume of sickle erythrocytes.^13^^,^^14^^,^^15^^,^^16^ The mean cell volume (MCV) increases during the first four to six weeks of HU treatment, and this is associated with clinical improvement. The increase in MCV occurs before the expansion of the F-cells (cells bearing HbF), and this is the hematological parameter that best correlates with a decrease in VOC episodes.^17^^,^^18^^,^^19^

Recently, a single center trial (LaSHS) reported the effect of prolonged administration of HU on morbidity and mortality among adult patients with SCD after 17 years of follow-up.^5^ The study suggested that administration of HU to adult SCD patients for a long period of time significantly reduced the incidence of acute and chronic complications of SCD and that it conferred a survival advantage.

Despite the growing body of evidence in the literature that HU therapy provides many benefits for sickle cell patients, this therapy is still underprescribed for many reasons (possible long-term side effects, low availability in emerging countries etc.). In the present study, we analyzed the clinical and laboratory effects of HU treatment among sickle cell patients followed up at our sickle cell centre in Ribeirão Preto, São Paulo, Brazil.

## OBJECTIVE

The objective of this study was to analyze the clinical and hematological effects of hydroxyurea on sickle patients followed up at our institution.

## METHODS

### Study design

This was a retrospective study conducted at our sickle cell centre. All the clinical and laboratory data were obtained from the patients’ medical records. This study was approved by the Ethics Committee of our institution (protocol number 2455/2004).

### Subjects

37 sickle cell patients (26 SS and 11 S-beta-thalassemia cases) followed up at our centre participated in the study. The inclusion criteria were: diagnosis of sickle cell anemia (SS) or S-beta-thalassemia with a moderate to severe phenotype requiring HU therapy. The indications for HU treatment were: VOC, priapism, pulmonary hypertension and acute chest syndrome (ACS). All the patients signed an informed consent statement before starting on HU.

The mean duration of HU treatment was 4.8 ± 3.2 years and the mean HU dose was 24.5 mg/kg/day ± 5.5 mg/kg/day. Some patients (9/37) did not tolerate the maximum dose prescribed (which started at 15 and could reach up to 35 mg/kg/day), due to neutropenia (neutrophils < 2,000/µl).

A total of 37 out of the 105 sickle cell patients taking HU who were being followed up at our institution met the above criteria and had complete laboratory analysis and clinical data available in the medical records. The patients’ median age was 23 years (range: 9 to 43) and the gender distribution was 19 males and 18 females.

The medication was provided by the Brazilian Ministry of Health to all sickle cell patients through a program within the Brazilian National Health System (Sistema Único de Saúde, SUS).

### Hematological parameters and HbF quantification

Peripheral blood samples were collected for quantification of hematological parameters (hemoglobin, MCV, white blood cells, WBC, neutrophils and platelet counts) before HU therapy and 3, 6 and 12 months after HU therapy had started, and at their last follow-up. HbF quantification was performed prior to the therapy and at least once a year.

The WBC and platelet counts, the hemoglobin concentration and the MCV were determined using the automated Coulter Gen S system 2 (Beckman-Coulter, CA, USA).

The HbF percentage was determined using the HPLC technique (Variant Express, Bio-Rad laboratories, Inc, Hercules, CA, USA) and the beta-thalassemia short program (Variant^TM^, Bio-Rad laboratories, Inc, Hercules, CA, USA).

### Clinical data

We reviewed the patient medical records, including the number of the following events: VOC, ACS, hospitalizations, infectious episodes and transfusions of RBC that occurred during the year before HU therapy and the first year of treatment.

### Statistical analysis

The hematological parameters were tested using analysis of variance (ANOVA) and the Tukey test if the samples had normal distribution. The Friedman/Dunn test was used if the samples did not have normal distribution. The clinical data and the percentage of HbF were analyzed using the Wilcoxon non-parametric test. The results were expressed as means and standard deviations or as medians (with range), respectively if they had normal distribution or did not. The statistical significance level was set at 5% (P *<* 0.05) for all analyses.

## RESULTS

### Hematological parameters

After one year of treatment, HU led to an increase of 0.7 g/dl in the hemoglobin concentration: from a mean of 8.3 g/dl ± 1.29 to 9.0 ± 1.4 g/dl (P = 0.0003). The MCV increased from 88.7 ± 13.5 to 104.8 ± 15 fl (P = 0.001), and HbF increased from 2.6 (0.16-8.47) to 19.8% (5.9-34.8, P < 0.0001). Additionally, HU treatment decreased the WBC from 11,800/µl (4,100-27,300) to 9,100/µl (1,000-17,900) (P < 0.0001), neutrophils from 6,200/µl (1,500-18,300) to 3,400/µl (700-11,900) (P = 0.001) and platelets from 459,000/µl (192,000-893,000) to 373,000/µl (109,000-870,000) (P = 0.0002). The reductions in those parameters could be seen after three months of therapy and lasted over the years (Table 1).

**Table 1.** Hematological parameters before hydroxyurea therapy and three, six and twelve months after therapy started

Parameters	Before HU M ? SD	3 months M ? SD	6 months M ? SD	12 months M ? SD	Last follow-up M ? SD
Hb (g/dl)	8.3 ? 1.29	8.6 ? 1.26	8.8 ? 1.28	9.0 ? 1.4	9.3 ? 1.4
MCV (fl)	88.7 ? 13.5	100.3 ? 13.3	102.3 ? 13.9	104.8 ? 15	114.2 ? 14.7
WBC (x10^3^/ml)	12.3 ? 3.9	9.6 ? 3.4	9.5 ? 3.1	9.6 ? 4.4	8.2 ? 3.5
Neutrophils (x10^3^/ml)	7.1 ? 3.6	4.9 ? 2.1	4.6 ? 1.8	5.1 ? 3.5	4.4 ? 2.4
Platelets (x10^3^ U/ml)	493 ? 161	390 ? 146	423 ? 161	396 ? 136	379 ? 124
HbF (%)	2.6 ? 2.1	*	*	19.8 ? 7.2	20.3 ? 8.6

M ± SD = mean ± standard deviation; Hb = hemoglobin; MCV = mean cell volume; WBC = white blood cells; HbF = fetal hemoglobin; *data not available.

### Clinical data analysis

**Figure 1.**
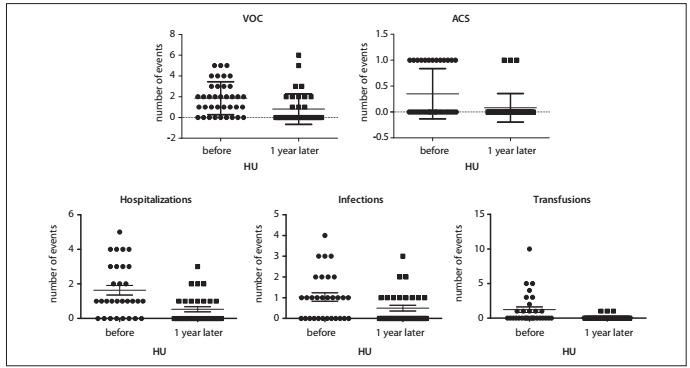
Change in the rates of vaso-occlusive crises (VOC) (mean 1.86 to 0.81 events/year, P = 0.0014), acute chest syndrome (ACS) (mean 0.35 ? 0.48 to 0.08 ? 0.28, P = 0.0045), hospitalizations (mean 1.63 ? 1.52 to 0.53 ? 0.82, P = 0.0013) and infections (mean 1.03) from before hydroxyurea (HU) therapy to one year later.

**Figure 2.**
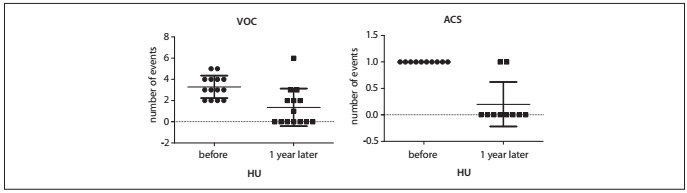
Change in the rate of vaso-occlusive crises (VOC) (mean 3.29 ? 1.07 to 1.36 ? 1.78, P = 0.01) among 14 patients for whom VOC was the indication to start hydroxyurea (HU). Change in the rate of acute chest syndrome (ACS) episodes (mean 1.0 to 0.2 ? 0.42, P = 0.006) among 10 patients for whom ACS was the indication to start HU. Comparisons were made from before to after one year of HU therapy.

After one year of treatment with HU, we observed reductions in the acute complications of sickle cell disease, such as VOCs, ACS and infections, as well as reductions in the need for transfusions and hospitalizations (Figure 1). The mean VOC rate dropped from 1.86 ± 1.58 events/year to 0.81 ± 1.47 events/year (P *=* 0.0014). The overall ACS rate also reduced (0.35 ± 0.48 to 0.08 ± 0.28, P *=* 0.0045), along with the numbers of hospitalizations (1.63 ± 1.52 to 0.53 ± 0.82, P *=* 0.0013), infections (1.03 ± 1.13 to 0.5 ± 0.78, P *=* 0.047) and RBC units transfused (1.23 ± 2.25 to 0.1 ± 0.3, P *=* 0.0051). We also analyzed separately the occurrences of VOC in 14 patients and ACS in 10 patients in which these events were the indication for starting HU (Figure 2). There were significant reductions in VOC (3.29 ± 1.07 to 1.36 ± 1.78, P *=* 0.01) and in ACS episodes (1.0 to 0.2 ± 0.42, P *=* 0.006) in these groups.

### Adverse events

We focused on myelotoxicity as the most important adverse effect of HU therapy. HU treatment can lead to decreased levels of hemoglobin (Hb < 6.0 g/dl), neutrophils (NE < 2,000/µl) and platelets (Pl < 80,000/µl) that can be life-threatening. Whenever these thresholds are reached, HU therapy is suspended and then restarted at a lower dose (MTD, maximum tolerated dose).

In this study, only neutropenia was observed, in 24.3% of the patients (9/37), and the HU dose was reduced for these patients. After adjusting the HU dose to the MTD, all these patients’ neutrophil counts recovered to greater than 2,000/µl.

## DISCUSSION

This study showed that these patients presented considerable clinical and hematological improvements, which is in accordance with previous reports.^17^^,^^19^ After three months of HU treatment, the patients presented a significant and persistent increase in MCV and reductions in WBC and platelet counts. These results are similar to what has been found in other studies (Table 2).^5^^,^^20^^,^^21^^,^^22^ HbF presented a remarkable increase up to the last follow-up (median from 2.6% to 19.8%). This marked response was greater than the response reported in the MSH study,^17^ probably due to the inclusion of children in our study, who are known to have a better HbF response under HU treatment.^10^^,^^18^ Among the studies shown in Table 2, Zimmerman et al.^21^ analyzed children and reported a similar percentage of HbF (19.7%), which was greater than the HbF reported from another study on children^20^ and in other two studies on adult patients.^5^^,^^17^ Another important factor could be the diverse genetic determinants of the response to HU among the subjects.^23^

**Table 2.** Hematological effects of hydroxyurea therapy reported in published clinical studies and the current study

Parameters	Current study	Kinneyet al.^20^	Zimmerman et al.^21^	Charache et al.^22^	Voskaridou et al.^5^
Number of patients	37	71	106	32	131
Average age (years)	23	9.8	10.3	27.6	33
Average dose (mg/kg/d)	24.5	25.6	25.9	21.3	20
Hb (g/dl)	9.3	9.1	9.5	9.7	9.5
MCV (fl)	114	102	107	117	97
WBC (x10^9^/l)	8.2	9.1	7.2	8.4	8.0
Neutrophils (x10^9^/l)	4.4	4.4	3.6	4.6	4.0
Platelets(x10^9^/l)	379	357	392	364	308
HbF (%)	19.8	16.3	19.7	15	17.4

Hb = hemoglobin; MCV = mean cell volume; WBC = white blood cells; HbF = fetal hemoglobin.

There was a marked reduction in significant clinical events during the HU treatment. After one year of therapy, the overall rate of VOC events dropped from 1.86 to 0.81/year. In the LaSHS study, this reduction was even greater (7.34 ± 6.5 to 0.05 ± 0.026), but the decreases in numbers of hospitalizations (2.11 ± 2.96 to 0.03 ± 0.19) and numbers of transfusions (1.53 ± 5.92 to 0.22 ± 0.95) were similar to the results presented in our study (1.63 ± 1.52 to 0.53 ± 0.82 and 1.23 ± 2.25 to 0.1 ± 0.3, respectively). There was also a five-fold reduction in occurrences of a second episode of ACS among the patients during HU therapy (1.0 to 0.2 ± 0.42, P *=* 0.006). In the MSH study, the incidence of ACS decreased in 50% of the patients and in the LaSHS study, the incidence dropped from 6.1% to 0.8% of the patients during therapy. In a Brazilian cohort of children, 224 patients were followed up for 10 years and the median treatment duration was 1.9 years (range: 1.2-6.1) with a median dose of 20 mg/kg/d (range: 15-28). There were significant reductions in hospitalization (67%), transfusions (36%) and emergency room visits (48.7%).^24^

Surprisingly, we noticed that there was a significant reduction in the frequency of infectious episodes (1.03 ± 1.13 to 0.5 ± 0.78, P *=* 0.047). Although HU reduced the number of circulating leukocytes and some patients took lower doses of HU because of transient neutropenia, the number of severe infectious episodes needing hospitalization was lower. The rate of infectious events was not addressed in most previous studies, but the LaSHS study reported two deaths caused by sepsis: one in a patient taking HU and the other in a patient without treatment.^5^

In this study, we did not analyze the impact of HU on mortality, but the LaSHS study suggested that administration of HU to adult sickle cell patients for a long period of time significantly reduced the incidence of acute and chronic complications of SCD and that these patients had a survival advantage.^5^ Patients who had HbF values greater than 2% had a 10-year probability of survival of 89%, compared with 53% among patients with HbF lower than 2%, thus showing the great impact of HbF on survival.^5^ In the Brazilian cohort, HU also improved survival. The cumulative survival rate at 17.9 years of age was 97.4% among patients taking HU, compared with 66.3% among those who were not treated.^24^

A quality of life (QoL) study conducted among sickle cell patients who participated in the MSH study showed that the benefit of HU was limited to the patients who maintained a high HbF response, compared with those with low HbF or on placebo, thus indicating that HbF response has a role not only in relation to survival, but also in relation to quality of life.^25^

Nowadays, most children with HbSS or S-beta^0^-thalassemia (93.9%) and nearly all children with HbSC or S-beta^+^-thalassemia (98.4%) live and reach adulthood in developed countries after the newborn screening program, with subsequent administration of prophylactic penicillin and immunization.^26^ In this cohort of children (DNC), ACS and multiple organ failure surpassed bacterial sepsis as the leading cause of death. According to the MSH and LaSHS studies, these two SCD complications can be prevented or reduced by HU therapy.^5^^,^^17^

In the BABY HUG study, very young children (aged 9-17 months) started on HU despite their clinical severity, and there were reductions in VOC, ACS, hospitalizations and transfusions, in comparison with placebo treatment, without any apparent increased risk of genotoxicity.^27^

Despite the growing body of evidence in the literature that HU therapy has many benefits for sickle cell patients, this therapy is still underused.^28^ After these recent studies, which have demonstrated remarkable benefits from HU treatment, this drug may become soon the standard of care for young patients with sickle cell disease.^28^^,^^29^ We hope that this study, in association with others that have recently been published,^24^^,^^26^^,^^30^ may encourage physicians, especially in emerging countries, to prescribe HU more often for their sickle cell patients.

## CONCLUSION

In this study, we showed that our patients had considerable clinical and hematological improvements with HU therapy that lasted up to the last evaluation.
